# Cystatin C deficiency suppresses tumor growth in a breast cancer model through decreased proliferation of tumor cells

**DOI:** 10.18632/oncotarget.17379

**Published:** 2017-04-24

**Authors:** Janja Završnik, Miha Butinar, Mojca Trstenjak Prebanda, Aleksander Krajnc, Robert Vidmar, Marko Fonović, Anders Grubb, Vito Turk, Boris Turk, Olga Vasiljeva

**Affiliations:** ^1^ Department of Biochemistry and Molecular and Structural Biology, Jozef Stefan Institute, SI-1000 Ljubljana, Slovenia; ^2^ Jozef Stefan International Postgraduate School, Sl-1000 Ljubljana, Slovenia; ^3^ Department of Clinical Chemistry, Laboratory Medicine, University Hospital, SE-22185 Lund, Sweden; ^4^ Centre of Excellence for Integrated Approaches in Chemistry and Biology of Proteins, SI-1000 Ljubljana, Slovenia; ^5^ Faculty of Chemistry and Chemical Technology, University of Ljubljana, SI-1000 Ljubljana, Slovenia; ^6^ Current address: CytomX Therapeutics, Inc., South San Francisco, CA 94080, USA

**Keywords:** cathepsin inhibitor, cystatin C, breast cancer, mouse model

## Abstract

Cysteine cathepsins are proteases that, in addition to their important physiological functions, have been associated with multiple pathologies, including cancer. Cystatin C (CstC) is a major endogenous inhibitor that regulates the extracellular activity of cysteine cathepsins. We investigated the role of cystatin C in mammary cancer using CstC knockout mice and a mouse model of breast cancer induced by expression of the polyoma middle T oncoprotein (PyMT) in the mammary epithelium. We showed that the ablation of CstC reduced the rate of mammary tumor growth. Notably, a decrease in the proliferation of CstC knockout PyMT tumor cells was demonstrated *ex vivo* and *in vitro*, indicating a role for this protease inhibitor in signaling pathways that control cell proliferation. An increase in phosphorylated p-38 was observed in CstC knockout tumors, suggesting a novel function for cystatin C in cancer development, independent of the TGF-β pathway. Moreover, proteomic analysis of the CstC wild-type and knockout PyMT primary cell secretomes revealed a decrease in the levels of 14-3-3 proteins in the secretome of knock-out cells, suggesting a novel link between cysteine cathepsins, cystatin C and 14-3-3 proteins in tumorigenesis, calling for further investigations.

## INTRODUCTION

Proteases play crucial roles in multiple steps of tumor progression and metastasis. Among the proteases associated with tumorigenesis and tumor progression, lysosomal cysteine proteases or cysteine cathepsins have gained major attention due to their high proteolytic potential under the more acidic conditions of the tumor microenvironment [1-3]. Although their main functions are associated with lysosomes, cysteine cathepsins can be secreted into the extracellular milieu, which is of particular importance in pathological processes, including cancer [4]. Numerous studies have shown a correlation between the increased proteolytic activity of cysteine cathepsins and neoplastic transformation, tumor invasion and metastasis through highly regulated and complex processes, including remodeling of the extracellular matrix, processing, or shedding of cell adhesion molecules, as well as growth factors and cytokines in the tumor microenvironment, leading to tumor spread [1-3, 5]. In different types of cancer, increased expression and proteolytic activity of lysosomal cathepsins have been correlated with poor cancer prognosis [6-10]. Moreover, cysteine cathepsins, in particular cathepsins B and L, were also found to promote migration and invasion of tumor cells [11-13]. In addition, they were suggested to participate in the proteolytic cascade activation [14, 15], which may all contribute to their tumor-promoting roles.

Protease activity is regulated by multiple mechanisms, with the most important being zymogen activation and inhibition by endogenous protein inhibitors [4, 16]. The best-known endogenous inhibitors of cysteine cathepsins are cystatins. Cystatins are further classified into three families: type 1 cystatins or stefins, which are intracellular inhibitors (stefins A and B); type 2 cystatins, which are extracellular inhibitors (cystatins C, D, E/M, F, S, SA and SN); and type 3 cystatins or kininogens, which are primarily found in blood plasma [17]. Type 2 cystatins contain a signal peptide that is responsible for secretion across the cell membrane, thereby representing an important regulatory mechanism for the extracellular activity of cysteine cathepsins. Cystatin C is the best-characterized type 2 cystatin and the most important extracellular inhibitor of cathepsins [18]. Cystatin C is a ubiquitously expressed secretory protein and a tight-binding inhibitor of lysosomal cysteine proteases, which inhibits their targets with nM (cathepsin B, asparaginyl endopeptidase (legumain)) to sub-pM (cathepsin L) affinity [17, 19, 20]. In addition to its role in physiological processes by controlling extracellular proteolysis via inhibition of cysteine proteases, cystatin C has been associated with different pathological conditions, such as neurodegenerative disorders, inflammatory and cardiovascular diseases, and cancer [21-23]. Increased levels of cystatin C in tumor tissues have been correlated with a favorable prognosis for cancer patients, whereas higher levels of cystatin C in body fluids have been associated with poor prognosis [23]. However, altered expression of cystatin C was reported in different types of cancer, although the levels varied depending on the cancer type. While reduced expression of cystatin C was observed in skin during the development of epithelial cancer [24], an increase in cystatin C expression was reported in ovarian cancer [25]. Interestingly, an inverse correlation between the cystatin C levels and tumor grade was reported in glioma, suggesting a role for cystatin C in the invasiveness of human glioblastoma cells [26]. In addition, higher levels of cystatin C have been detected in the cyst fluid of patients with ovarian cancer [27] and in the sera of patients with lung [28] or colorectal cancer [29]. Moreover, cystatin C was identified as a novel antagonist of transforming growth factor β (TGF-β) type II receptor (TβR-II), which prevents the binding of this receptor to TGF-β, thereby inhibiting TGF-β signaling in normal and malignant cells [30], suggesting a role for cystatin C apart from the inhibition of cathepsins. Taken together, the precise role for cystatin C in tumor progression is not fully elucidated and remains controversial. Therefore, to reveal the function of cystatin C at different stages of tumor progression and metastasis *in vivo*, we employed a mammary tumor virus (MMTV) Polyoma Middle T (PyMT) transgenic mouse model of mammary cancer [31] and crossed it with cystatin C-deficient mice [32]. The data demonstrated that the depletion of cystatin C in the PyMT breast cancer model reduced tumor growth and proliferation, suggesting a novel function for cystatin C in cancer development and progression.

## RESULTS

### Generation and characterization of PyMT transgenic mice with CstC deficiency

CstC-deficient mice (CstC-/-) were backcrossed to the FVB/N genetic background for more than 10 generations. These mice did not show any gross phenotype at 14 weeks of age and were further crossed with FVB/PyMT transgenic mice, resulting in two cohorts of female mice that were hemizygous for the PyMT transgene: PyMT;CstC+/+ and PyMT;CstC-/-.

**Figure 1:**
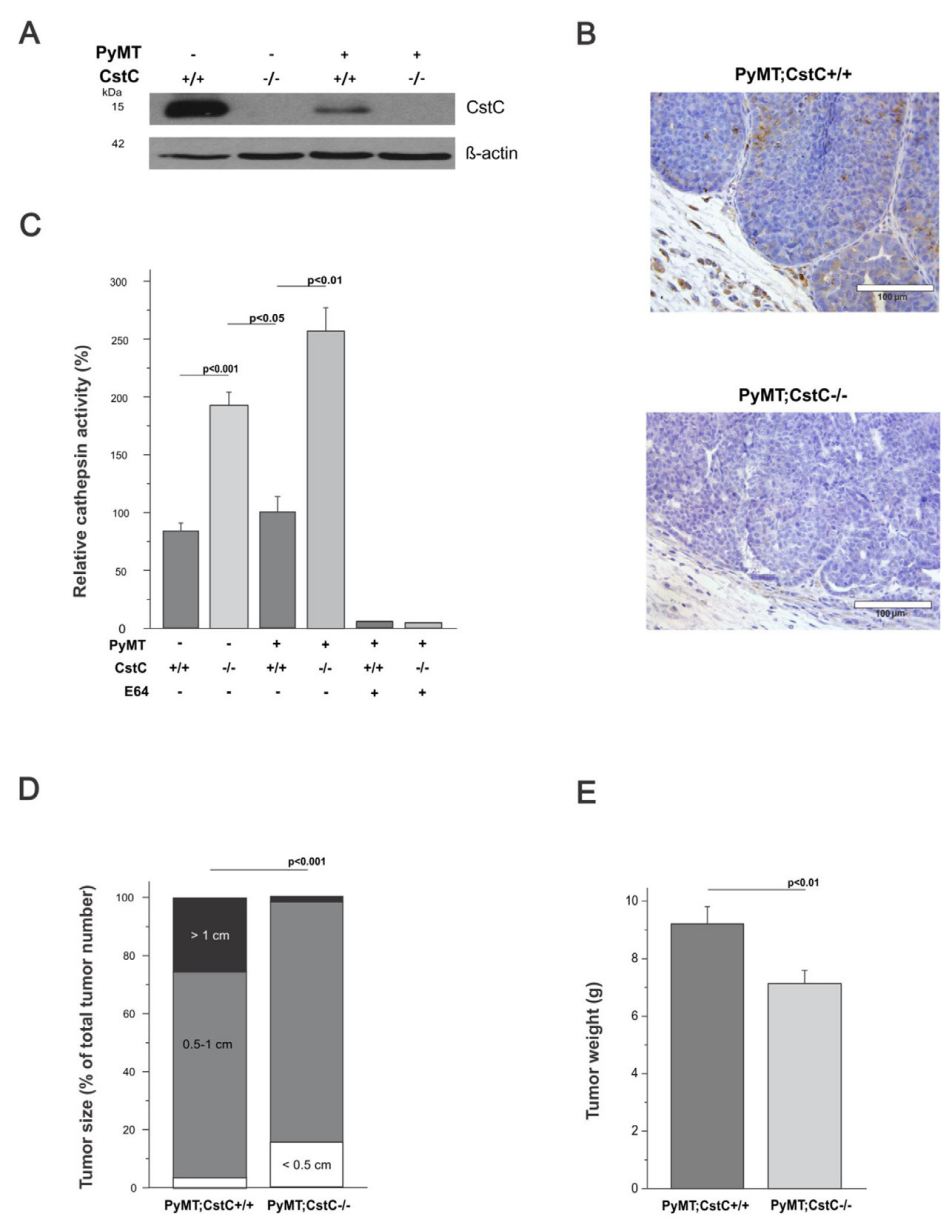
Suppression of PyMT-induced tumor growth in cystatin C deficient female mice **(A)** Expression of cystatin C evaluated by Western blot analysis in normal mammary glands (FVB;CstC+/+, FVB;CstC-/-) and PyMT-induced mammary carcinomas (PyMT;CstC+/+, PyMT;CstC-/-) at 14 weeks of age. β-actin was used as the internal loading control. **(B)** Detection of cystatin C by immunohistochemistry staining of PyMT;CstC+/+ and PyMT;CstC-/- tumor sections. Scale bar, 100 μm. **(C)** Relative cathepsin activity determined in homogenates of tumors (PyMT;CstC+/+ n=4 and PyMT;CstC-/- n=4) and mammary glands (FVB;CstC+/+ n=4, FVB;CstC-/- n=4). Activity was measured as Z-Phe-Arg-AMC hydrolysis in the presence or absence of E64. Statistics were analyzed using Student`s t-test. **(D)** Size of mammary tumors 30 days after first detection of the individual tumor node. The first 2 individual mammary tumors in each female were analyzed; thus, 35 and 33 individual tumors were included in the PyMT;CstC+/+ and PyMT;CstC-/- groups, respectively. The Chi-square test was used for statistical analyses. P<0.001. **(E)** The cumulative tumor weight per mouse for all mammary tumors was calculated for PyMT;CstC+/+ (n=23) and PyMT;CstC-/- (n=26) mice at 14 weeks of age. Statistics were analyzed using Student’s t-test. P<0.01.

First, the expression level of CstC was analyzed in PyMT tumor and normal mammary glands of 14-week-old mice using Western blotting. Interestingly, while CstC was highly expressed in normal mammary gland tissue, its protein level was substantially reduced in the tumor tissue (Figure 1A). Furthermore, immunohistochemical staining for CstC in PyMT tumors revealed that CstC was predominantly localized in the tumor stroma and at the edge of tumor nodes, and much less expression was observed in the tumor core, whereas homogeneous staining of CstC was observed in mammary glands (Figure 1B and Supplementary Figure 1). This observation suggests the down-regulation of CstC expression during the malignant transformation of the mammary epithelium, consistent with previous reports on decreased CstC expression in patient tumor tissues [33, 34]. Furthermore, these data suggest that due to the reduced cystatin C levels, cathepsins may escape internal control, resulting in elevated extracellular cathepsin activities.

To evaluate this idea, we next investigated the effect of CstC ablation on the activity of cysteine cathepsins in normal mammary glands and mammary tumors by monitoring the hydrolysis of the general cathepsin fluorogenic substrate Z-Phe-Arg-AMC in the lysates of tumors and normal mammary tissues of both genotypes (Figure 1C). A significant increase in the hydrolysis of Z-Phe-Arg-AMC was observed in both CstC-deficient tissues compared to wild-type controls, consistent with the protective role of CstC. Moreover, Z-Phe-Arg-AMC hydrolysis was much higher in CstC-deficient PyMT tumor lysates compared to CstC-deficient normal mammary tissue, consistent with the elevated cathepsin activity in malignant tissue. Consistently, addition of human recombinant CstC to the tumor lysates significantly decreased cathepsin activity (Supplementary Figure 2).

### Progression and metastasis of CstC depleted PymT-induced mammary carcinomas

To further investigate the role of CstC ablation on tumor development and growth, the volumes of the first two palpable mammary tumors were measured at 30 days after the first tumor detection and grouped according to their diameter into three categories: small (<5 mm), medium (5-10 mm) and large (>10 mm) (Figure 1D). Surprisingly, PyMT;CstC-/- tumors were significantly smaller compared to tumors from PyMT;CstC+/+ mice. This observation was confirmed *ex vivo*, where the cumulative weight of all 10 mammary tumors from mice at the age of 14 weeks was measured (Figure 1E). The weight of tumors from PyMT;CstC-/- mice was 7.1 g, which is 23 % lower compared to tumors from PyMT;CstC+/+ mice, which had an average tumor weight of 9.2 g. These findings further suggest that CstC exhibits pro-tumorigenic activity in the PyMT mammary cancer model.

**Figure 2:**
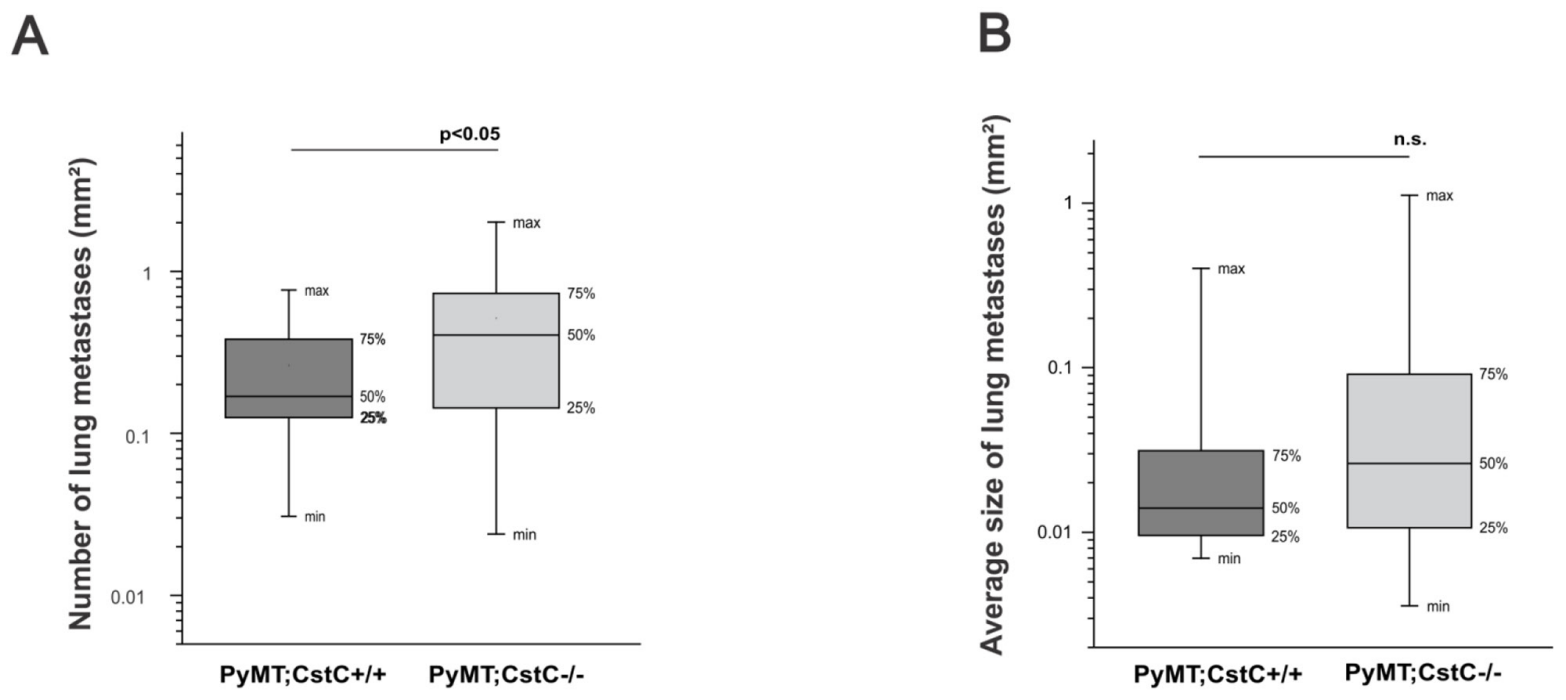
Effect of cystatin C deficiency on pulmonary metastases of PyMT mammary tumors Histomorphometric analysis of **(A)** number and **(B)** average size of lung metastasis in PyMT;CstC+/+ (n=18) and PyMT;CstC-/- (n=22) female mice at age of 14 weeks. Statistics were analyzed using Student’s t-test.

We next addressed how CstC depletion affects metastasis development and growth. In the MMTV;PyMT mouse model, pulmonary metastases occur in most animals at the age of 14 weeks. We therefore assessed the number and cumulative size of pulmonary metastases using computer-assisted histomorphometry in sequential histological sections of lungs from both experimental groups of mice at the age of 14 weeks. Compared to the PyMT;CstC+/+ group, the number of lung metastases in the PyMT;CstC-/- group calculated per 1 mm^2^ of lung tissue was significantly higher (0.51 in PyMT;CstC-/-, 0.26 in PyMT;CstC+/+) (Figure 2A). However, although there was a trend towards an increase in the average size of lung metastases in CstC-deficient PyMT mice, no statistically significant difference was detected between the two cohorts of mice (Figure 2B).

### Effect of CstC ablation on tumor cell proliferation, cell death and angiogenesis

**Figure 3:**
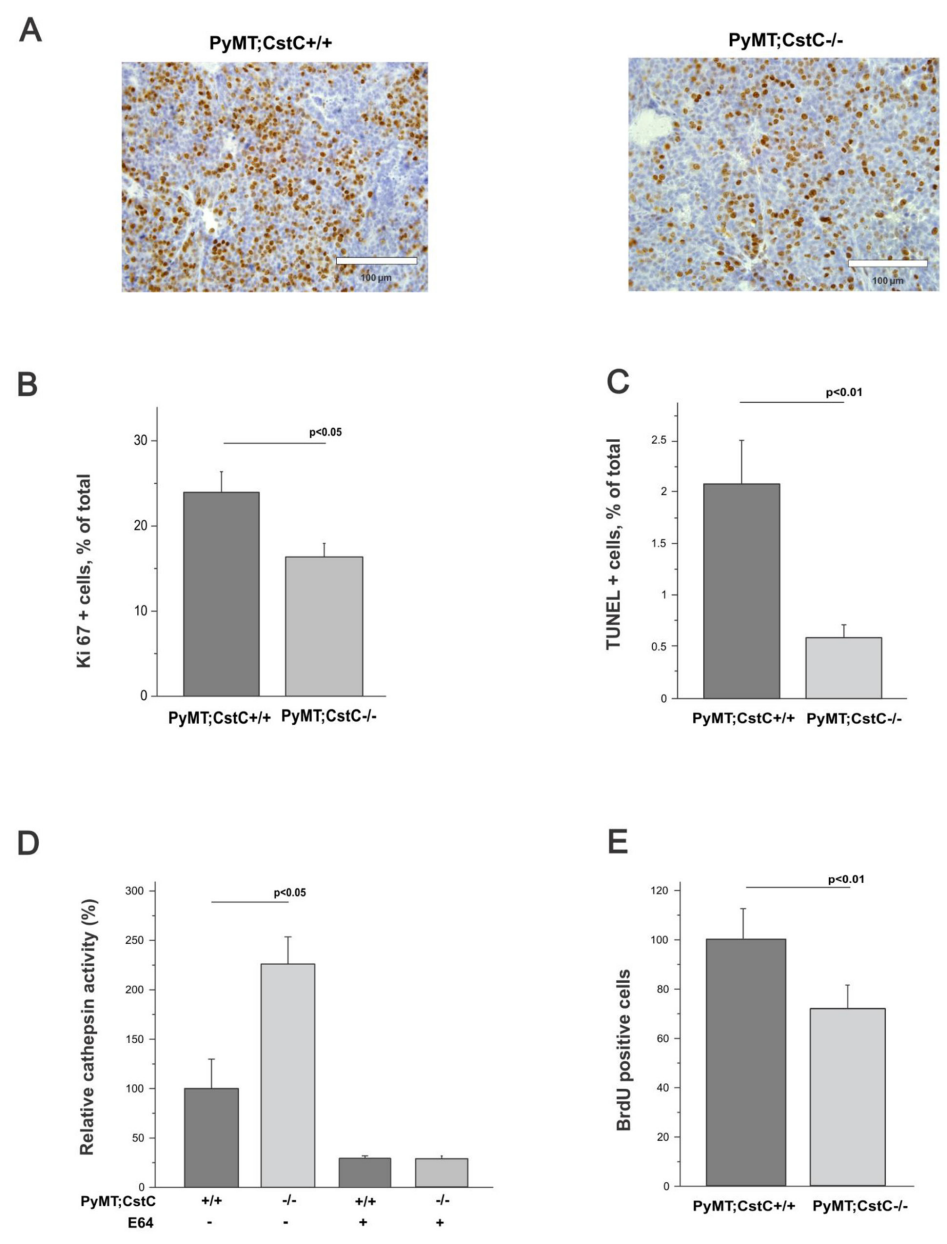
Effect of cystatin C deficiency on proliferation, cell death and vessel density **(A)** Cell proliferation in primary tumors determined through immunodetection of Ki67 in PyMT;CstC+/+ and PyMT;CstC-/- mice at 14 weeks of age. Representative images for each genotype are shown under 40x magnification. **(B)** Quantification of Ki67-positive cells as a percentage of total cells in PyMT;CstC+/+ (n=11) and PyMT;CstC-/- (n=12) tumors. Ki67 index was calculated from 20 high-power fields per tumor through computer-assisted data analysis using ImageJ software. Statistics were analyzed using Student’s t-test. P<0.05. **(C)** Quantification of TUNEL-positive cells as a percentage of total cells from each of PyMT;CstC+/+ (n=9) and PyMT;CstC-/- (n=9) tumors of 14 weeks old mice. TUNEL+ index was calculated from 10 high-power fields per tumor by computer-assisted data analysis using ImageJ software. Statistics were analyzed using Student’s t-test. P<0.01. **(D)** Relative cathepsin activity determined in cell culture medium of primary tumor cells (PyMT;CstC +/+, n=3, PyMT;CstC-/-, n=3). Activity was measured as Z-Phe-Arg-AMC hydrolysis in the presence or absence of E64. Statistics were analyzed using Student’s t-test. **(E)** Percentage of BrdU incorporation into nuclei of primary tumor cells (PyMT;CstC+/+, n=5, PyMT;CstC-/-, n=5). Statistics were analyzed using Student’s t-test. P<0.01.

We next analyzed whether CstC ablation affects major processes associated with tumor progression, such as tumor cell proliferation, cell death and angiogenesis in late-stage primary PyMT tumors. First, cell proliferation of mammary tumors isolated from PyMT;CstC-/- and PyMT;CstC+/+ mice was evaluated by immunohistochemical detection of the proliferating factor Ki67 (Figure 3A). Consistent with the reduced size of PyMT;CstC-/- tumors (Figure 1E), the Ki67 proliferation index was reduced in PyMT;CstC-/- tumors (16.4 %) compared to PyMT;CstC+/+ tumors (24 %) (Figure 3B). In addition to the lower rate of tumor cell proliferation, reduced tumor size may also result from increased cell death or the reduced vascularization of tumors. Therefore, we next quantified the cell death of cancer cells in primary PyMT tumors using terminal dUTP nick-end labeling (TUNEL) staining as a measure of DNA fragmentation, which is directly related to apoptosis. Although the fraction of TUNEL-positive cells in PyMT primary tumors was low (2 % in average), apoptotic cell death was lower in CstC-deficient tumors compared to wild-type controls (Figure 3C). This was confirmed by quantification of active caspase 3 in primary PyMT tumors from CstC-deficient mice compared to wild-type controls (Supplementary Figure 3). We next evaluated the potential impact of CstC ablation on the vascularization of PyMT tumors by immunofluorescence staining of endothelial cells in primary tumor sections by measuring the levels of CD31, a known marker of angiogenesis. Notably, the quantification of CD31+ cells revealed no genotype-dependent differences in the mean vessel density between PyMT; CstC-/- and PyMT; CstC+/+ tumors (Supplementary Figure 4A and 4B). Taken together, these data suggest a role for cystatin C in tumor cell proliferation, but not in apoptotic cell death or angiogenesis, potentially explaining the significant reduction of PyMT tumors in CstC-deficient mice.

To better understand the role of cystatin C in tumor cell proliferation at the molecular level, we next investigated the level of protease activity and the proliferation rate of the primary PyMT;CstC-/- and PyMT;CstC+/+ tumor cells. First, the activities of the cathepsins were measured in conditioned media of isolated primary PyMT tumor cells of both CstC genotypes with fluorogenic substrate Z-Phe-Arg-AMC. A significant increase in substrate cleavage was detected in PyMT;CstC-/- tumor cells compared to wild-type PyMT tumor cells, providing evidence for the presence of extracellular cysteine cathepsins in PyMT cells (Figure 3D). Furthermore, the BrdU assay performed on primary PyMT tumor cells revealed a significant decrease in the proliferation index of PyMT;CstC-/- tumor cells (approximately 30 %) compared to PyMT;CstC+/+ tumor cells (Figure 3E), thereby confirming the Ki67 proliferation data obtained from the analysis of the primary tumor tissues.

**Figure 4:**
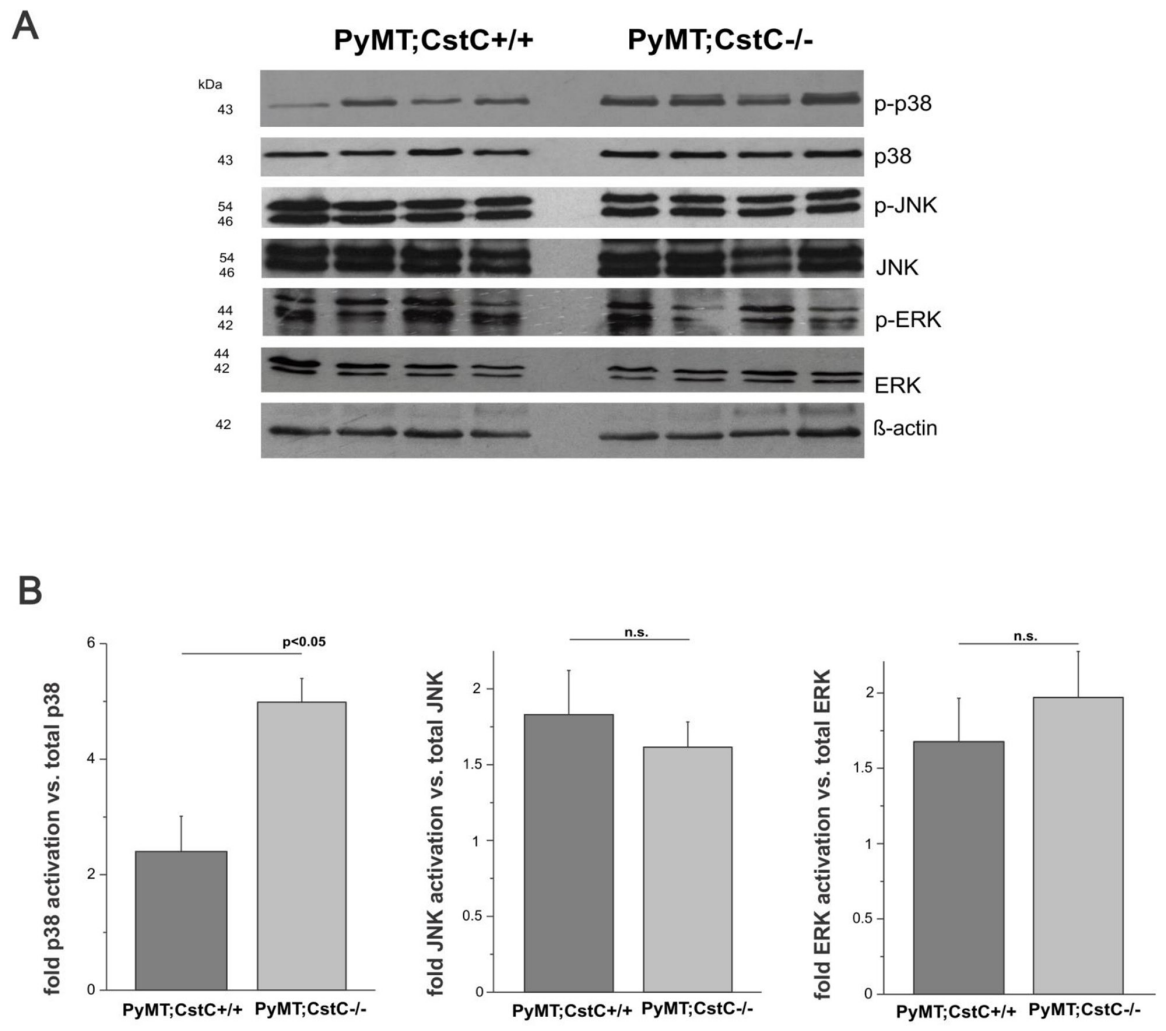
Phosphorylation of MAPK family **(A)** Western blot analysis of phosphorylated p38, total p38, phosphorylated JNK and total JNK, phosphorylated ERK and total ERK in lysates of representative tumors from 4 PyMT;CstC+/+ and 4 PyMT;CstC-/- mice. β-actin served as a loading control. **(B)** Quantification of phosphorylated p38 MAPK, JNK and ERK signals by densitometry analysis using ImageJ program. The results represent the mean value of the fold increase ± S.E. relative to the amount of total p38 MAPK, JNK and ERK proteins, respectively. The experiment was performed on 4 different biological samples per genotype. Statistics were analyzed using Student’s t-test. P<0.05.

However, because it is difficult to explain the effect on tumor cell proliferation solely by cathepsin inhibition, we next addressed the potential role for CstC as an antagonist of transforming growth factor β (TGF-β) signaling [35]. We therefore examined whether the absence of CstC expression affected the activation of mitogen-activated protein kinases (MAPKs), p38, JNK and ERK, which are associated with the TGF-β signaling [36, 37], by Western blot analysis of primary tumor lysates to monitor the activation status of these signaling molecules (Figure 4A). Notably, the phosphorylation and activation of p38 MAPK was increased in PyMT tumors deficient for CstC compared to their control counterparts (Figure 4B), supporting the idea that CstC acts as an antagonist of transforming growth factor β (TGF-β) signaling in normal and malignant cells [30]. However, whether the activation of p38 signaling pathways in PyMT may induce down regulation of tumor cell proliferation remains unknown.

### Impact of CstC from mouse breast cancer cells on proliferation and growth of PyMT tumors

**Figure 5:**
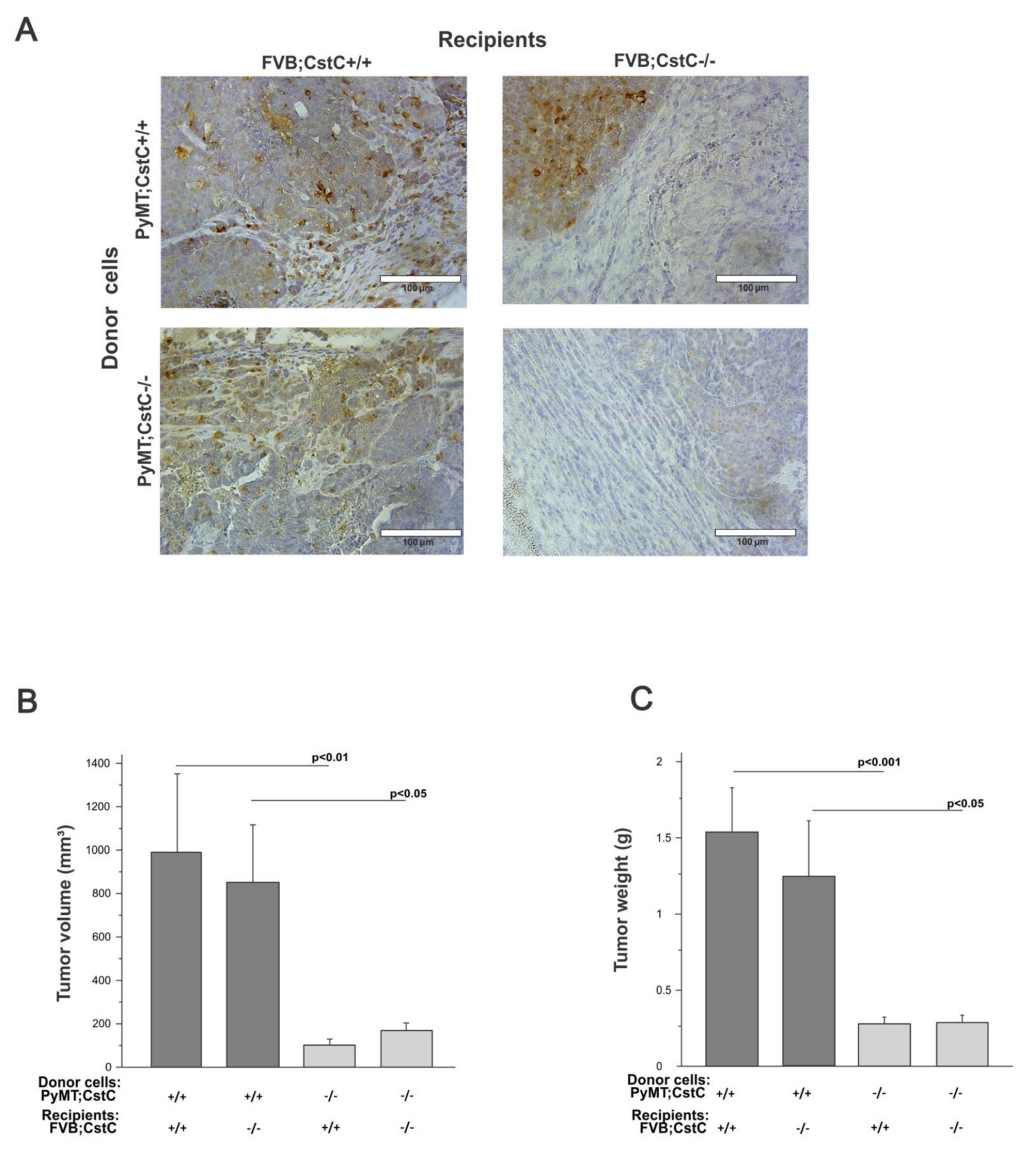
Orthotopic transplantation through intra-mammary injection of PyMT cells of both cystatin C genotypes into congenic recipients of both cystatin C genotypes **(A)** Detection of cystatin C by immunohistochemistry staining of orthotopical transplanted tumor sections. Scale bar, 100 μm. **(B)** Tumor volume of tumors from PyMT;CstC+/+ and PyMT;CstC-/- tumor cells, injected into FVB;CstC+/+ or FVB;CstC-/- female mice (number of each group: ≥7). Statistics were analyzed using Student’s t-test. **(C)** Tumor weight of tumors from PyMT;CstC+/+ and PyMT;CstC-/- tumor cells, injected into FVB;CstC+/+ or FVB;CstC-/- female mice (number of each group: ≥7). Statistics were analyzed using Student’s t-test.

Another factor that can critically affect the development and progression of cancer is the crosstalk between tumor cells and the tumor stroma [38, 39]. Since CstC is expressed and present in both the tumor cells and stromal compartments (Figure 1B) of primary PyMT tumors, we developed an orthotopic tumor model, in which primary PyMT;CstC-/- and PyMT;CstC+/+ syngeneic mouse breast cancer cells were implanted into the mammary fat pad of female recipient mice of both CstC genotypes (Figure 5A). Starting at two weeks post implantation, the tumor diameters were measured three times per week during a 20-day observation period. Notably, tumors from mice originating from PyMT;CstC-/- tumor cells were significantly smaller and their weight was significantly lower than that of PyMT;CstC+/+ tumor cell donors, independent of the recipient CstC genotype (Figure 5B and 5C). Taken together, this result suggests that the reduced proliferation of PyMT;CstC-/- tumor cells could result in decreased tumor growth *in vivo*, consistent with the findings on the inhibition of tumor progression in transgenic PyMT mouse breast cancer model deficient for CstC.

**Figure 6:**
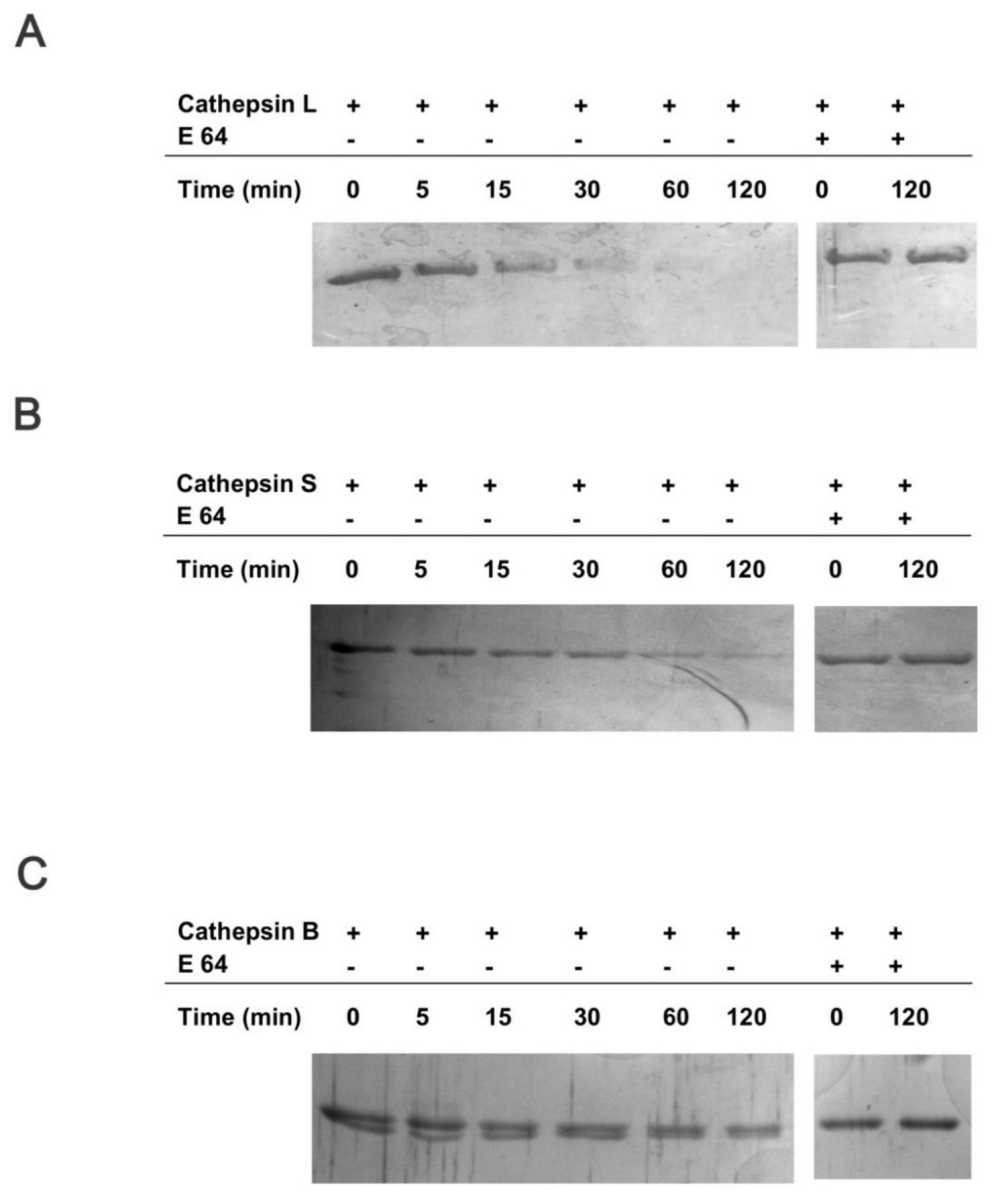
14-3-3β protein is a target substrate for cathepsins L, S and B *In vitro* assessment of proteolytic cleavage of 14-3-3β protein by cathepsins L, S and B. Human 14-3-3β protein was incubated at pH 6 and 37 °C with cathepsins **(A)** L, **(B)** S and **(C)** B and the level of 14-3-3β protein was evaluated at different time points in the presence or absence of E64.

Collectively, these data suggest that CstC from tumor cells, and not the stroma, plays an important role in the observed tumor growth phenotype of both transgenic and orthotopic implantation mouse models. To further investigate the potential mechanism of CstC ablation on the decreased proliferation of PyMT tumor cells, we performed a proteomic analysis of the PyMT tumor cell secretome. Conditioned media samples from PyMT;CstC-/- and PyMT;CstC+/+ tumor cells isolated from tumor-bearing mice were analyzed using mass spectrometry. The relative quantification of secreted proteins by spectral counting confirmed that CtsC is secreted from PyMT tumor cells in addition to several cysteine cathepsins, such as cathepsins B, Z(X), and L1(V) and asparaginyl endopeptidase [40] (Table 1). Whereas the lack of CstC was confirmed in PyMT;CstC-/- cell-conditioned medium, no difference in cysteine cathepsin levels was detected in cells of both genotypes. The latter funding was also confirmed by evaluation of their mRNA, protein expression and intracellular activity level (Supplementary Figures 5 and 6). Notably, the largest difference between the CstC knockout and wild-type tumor cell secretomes was observed for the 14-3-3 proteins, which were decreased in knockout animals (Table 1). 14-3-3 adaptor proteins have a wide range of roles in cell signaling and are also known to be present in the extracellular environment where they regulate biological activities with relevance for tissue homeostasis and diseases, such as cancer [41]. Therefore, proteolysis of 14-3-3 proteins could represent an alternative mechanism for their down-regulation that would affect their function, including their reported role in stimulation of matrix metalloproteinase (MMP) activity [42]. To test this idea we incubated recombinant 14-3-3β protein with active cathepsins B, L, and S *in vitro* under defined conditions and analyzed the reaction at different time points. The data revealed that 14-3-3β protein can be specifically cleaved by cathepsins L and S, and to a lesser extent by cathepsin B (Figure 6), suggesting a novel mechanism for cysteine cathepsin’s tumor-suppressive role.

**Table 1:** List of proteins identified in conditioned media from primary PyMT tumor cells with differential expression of CstC

Protein name	Uniprot accession	Number of peptides	MS/MS count		Fold difference
			PyMT;	PyMT;	
			CstC+/+	CstC-/-	
Cystatin C	P21460	9	46	0	
Cathepsin B	P10605	17	42	44	0.96
Cathepsin L(V)	P06797	15	34	36	0.94
Cathepsin Z(X)	Q9WUU7	12	16	18	0.89
Legumain	O89017	12	9	9	1.00
14-3-3 protein beta/alpha	Q9CQV8	15	17	9	1.89
14-3-3 protein epsilon	P62259	24	123	74	1.66
14-3-3 protein eta	P68510	18	19	10	1.90
14-3-3 protein gamma	P61982	20	38	24	1.58
14-3-3 protein sigma	O70456	21	46	20	2.30
14-3-3 protein theta	P68254	19	27	14	1.93
14-3-3 protein zeta/delta	P63101	21	64	42	1.52

Conditioned media of primary PyMT tumor cells from 4 different transgenic mice of each genotype were used.

## DISCUSSION

Protease activity is tightly regulated within cells and organisms, and inappropriate proteolysis can play a major role in the development of pathological conditions, such as cancer [43]. To better understand the function of proteases and successfully develop novel therapeutic strategies, it is necessary to understand the role of proteases in the complex sequence of events leading to the development of pathology and its progression. One of the major regulating mechanisms of proteases is the inhibition of their activity by endogenous protein inhibitors [16, 17]. The expression of proteases and their inhibitors in tumors is frequently dysregulated, which is often based on complex interactions between tumor cells and their microenvironment [44] and metabolic changes in the tumor microenvironment such as hypoxia and acidosis [45]. In the present study, we evaluated the impact of the depletion of the extracellular cysteine cathepsin inhibitor cystatin C on the development and progression of breast cancer using a PyMT mammary tumor model. Cysteine cathepsins, as well as legumain, a cysteine endopeptidase of the asparaginyl endopeptidase family, are reportedly secreted into the extracellular milieu by tumor or tumor associated cells of the tumor microenvironment, where they promote tumor progression and metastasis through complex processes involving cleavage, processing, or shedding of signal molecules and extracellular matrix proteins [10, 46]. Notably, while the ablation of CstC expression in PyMT transgenic mice increased the activity of cysteine cathepsins, as detected in the conditioned medium of primary PyMT tumor cells, a significant reduction in tumor growth and tumor volume was detected in PyMT;CstC-/- mice compared to CstC wild-type PyMT transgenic mice.

Although this finding is a bit surprising, as cathepsins were long believed to be tumor promoting proteases, it is now clear that they can also play “antagonistic” tumor-promoting roles, depending on the tissue and the type of cancer studied [10]. For example, the ablation of cathepsin B (Ctsb) in the PyMT model resulted in a significant decrease in mammary tumor growth and volume [2, 47]. In support of the pro-tumorigenic function of cathepsin B, the transgenic expression of human Ctsb promoted progression and metastasis in the same mammary cancer transgenic model [48]. Interestingly, although the depletion of another lysosomal cysteine protease, cathepsin Z (Ctsz; also called cathepsin X/P), significantly increased the sizes of PyMT tumors on weeks 10 and 14, profound synergistic anticancer effects were observed in mice after the combined loss of Ctsb and Ctsz [3]. Similarly, the ablation of cathepsins B, L, H, S or Z substantially reduced tumorigenesis in the pancreatic islet tumor model, although their individual roles clearly differ [49-51]. However, the deficiency of cathepsin L (Ctsl) enhanced tumor progression and metastasis in the K14–HPV16 transgenic mouse model of epidermal carcinogenesis through modulation of protein kinase B/Akt and mitogen-activated protein kinase pathways [52]. The removal of the cathepsin C gene in the same tumor model had a tumor suppressing effect, whereas the ablation of cathepsin B had no effect [53]. In addition, the functions of multiple cathepsins as sheddases that cleave extracellular receptors, such as plexins and EGFR, were recently uncovered, further supporting their tumor suppressing roles [54].

Taken together, these observations indicate a potential dual role for cysteine proteases in tumor progression. Consistently, a decrease in PyMT tumor progression was observed upon depletion of stefin B, the general cytosolic cysteine cathepsin inhibitor [55]. In contrast to CstC, stefin B is an intracellular inhibitor that plays an important role in the development of resistance of cells to oxidative stress and reactive oxygen species (ROS)-mediated apoptosis [55, 56]. However, whereas no change in the proliferation of PyMT tumor cells *in vivo* and *in vitro* was detected in StfB-/- PyMT mice, a significant decrease in the Ki67 and BrdU proliferation index was measured in CstC knock-out PyMT primary tumors and isolated PyMT cells, indicating a potential role for cystatin C in the regulation of the proliferative activity of tumor cells. This hypothesis was confirmed by the decreased tumor growth in an orthotopic transplantation breast cancer model, providing additional evidence that the role of CstC is associated with tumor cells and is not related to stromal cell effects. Cystatin C was identified as a novel TGF-β type II receptor (TβR-II) antagonist that prevents the binding of this receptor to TGF-β and, consequently, inhibits TGF-β signaling in normal and malignant cells [30]. Subsequently, this observation was confirmed *in vivo*, demonstrating a reduction in tumor growth and metastasis upon use of the 4T1 mouse breast cancer cells overexpressing CstC [57]. Interestingly, while the increase in the phosphorylation and activation of p38 MAPK was detected in PyMT breast cancer models deficient for CstC, the corresponding increase in tumor cells proliferation, growth or metastasis was not observed. This finding suggests that the expression level of CstC was not sufficiently high enough in the PyMT model to overcome the threshold needed for phosphorylated p38 MAPK regulation of the expression of downstream genes [58]. This idea is supported by the significant decrease in CstC expression detected in the PyMT tumors compared to normal breast tissue (Figure 1A ), providing evidence for the downregulation of CstC in the PyMT mammary tumor model. This observation is consistent with previously reported evidence of the decreased cystatin C expression in prostate cancer [34] and breast cancer [59] as well as during malignant progression in skin cancer [24]. Although elevated expression of another type 2 cystatin, cystatin M, was also found to be associated with malignant progression [60, 61], its expression was not detected by proteomic analysis in either PyMT cell lysates or conditioned tumor cell media, thereby excluding the possibility of the compensatory effect.

However, using a mass spectrometry-based approach, we identified a potential alternative mechanism for the regulation of the proliferative activity of tumor cells in the PyMT mammary cancer model through proteolytic degradation of 14-3-3 proteins. The 14-3-3 proteins comprise a family of highly conserved dimeric proteins in eukaryotic organisms and have seven isoforms in humans that play crucial roles in regulating multiple cellular processes, including cell growth, survival and differentiation [62]. Increased expression of 14-3-3 proteins was associated with tumor progression of various malignancies [63]. Furthermore, the association of 14-3-3 proteins with PyMT oncogene products has previously been reported and demonstrated as being dependent on phosphorylation at serine 257 [64, 65]. Among the 14-3-3 proteins, especially the 14-3-3β protein plays a vital role in tumor growth and progression. As such, 14-3-3β isoform expression was up-regulated in multiple types of cancer, such as Kaposi’s sarcoma [66, 67], gastric cancer [68], papillary thyroid carcinoma [69], hepatocellular carcinoma [70, 71], lung cancer [72] and gliomas [73]. Studies have shown that 14-3-3β overexpression in primary hepatocellular carcinoma predicts a higher 5-year incidence of subsequent metastasis and correlates with a worse 5-year overall survival [70]. The extracellular function of 14-3-3β protein was demonstrated in human lung epithelial cells, leading to enhanced MMP-1 expression [74]. Although no MMP-1a, the mouse ortholog of human MMP1, was detected in the secretome of PyMT cells, other molecular pathways can be activated by the extracellular form of the 14-3-3β protein, promoting the progression of mammary cancer in the PyMT breast cancer model. Furthermore, 14-3-3β protein was shown to regulate the transcriptional activity of ERα in a ligand-dependent manner and to increases the breast cancer cell proliferation [75]. It was shown that overexpression of the 14-3-3ζ isoform promoted mammary tumorigenesis and progression via enhanced neoplastic cell proliferation in a transgenic breast cancer model [76]. Moreover overexpression of this isoform of the 14-3-3 protein correlated with the advanced stage breast cancer and was determined to be an independent prognostic factor for reduced disease-free breast cancer survival [77]. Importantly, we have demonstrated that cathepsins L, S and B can cleave the 14-3-3β protein, suggesting that CstC might protect the 14-3-3 proteins from degradation by cysteine cathepsins, thereby providing an additional explanation for the potential tumor suppressor role of these proteases.

Taken together, these results show that CstC depletion negatively affects tumor cell proliferation, resulting in the decreased growth of tumors in an orthotopic mouse model of breast cancer as a consequence of the complex interplay between cysteine cathepsins activity, cystatin C, 14-3-3 proteins and TGF-beta signaling pathways. Currently, most studies focusing on the inhibition of protease activity have examined the application of protease inhibitors and/or protease knockout models. However, few research programs are aimed at a better understanding of the excessive protease activities associated with an imbalance of the activities of endogenous inhibitors. Thus, despite the well-known role of cysteine cathepsin as promoters of tumor progression, the present study on the ablation their major extracellular inhibitor enabled us to uncover novel functions of cysteine cathepsins in the tumor microenvironment that should be considered when designing novel protease inhibition strategies for cancer treatment.

## MATERIALS AND METHODS

### Animals

Mice were used in accordance with the Admini-stration of the Republic of Slovenia for food safety, veterinary and plant protection. Procedures for animal care and experiments were in accordance with the “Guide for the Care and Use in Laboratory Animals”. Cystatin C (CstC-/-) knockout mice were established on C57BL/6 strain in 1999 [32]. The CstC-/- knockout mice on C57BL/6 strain were kindly provided by dr. Anders Grubb (Lund University, Lund, Sweden). CstC-/- mice were backcrossed for more than 10 generations to the FVB transgenic mouse strain expressing PyMT under the control of the MMTV long-terminal repeat promoter (FVB/N-TgN (MMTVPyVT)634-Mul). After the intercross, wild type and CstC-deficient tumor bearing mice were named PyMT;CstC+/+ and PyMT;CstC-/-, respectively. Maintenance and breeding of the animals used in this study were performed in accordance with Slovene law for animal protection as published in May 2013.

### Tumor progression study

At 30 days of age, female mice were examined three times a week for the development of mammary tumors by palpation in a genotype-blinded fashion, and 30 days after detection of the first tumors, the diameters of the tumors were measured with a digital caliper. At the age of 14 weeks, the mice were euthanized, and all 10 tumors were excised, measured and weighted.

### Immunohistochemistry and immunofluorescence staining

Left thoracic tumors of PyMT;CstC-/- and PyMT; CstC+/+ mice were collected and fixed in 10 % Neutral-buffered Formalin overnight and then processed for paraffin embedding. Formalin fixed, paraffin embedded samples of tumors were sectioned (5 µm) and affixed to glass slides. The slides were deparaffinized and rehydrated through graded alcohol to distilled water, followed by antigen retrieval with citrate buffer, pH 6.0. Endogenous peroxidase was quenched with 0.3 % Hydrogen Peroxide. The sections were stained with antibodies against CstC (Abcam, Cambridge, UK; ab 109508, rabbit monoclonal, dilution 1:500), Ki67 (Dako, Carpinteria, CA, USA; M724901, rabbit monoclonal, dilution 1:50), CD31 (BD Pharmingen, San Diego, CA, USA; 550274, rabbit monoclonal, dilution 1:50) and active caspase 3 (Abcam, Cambridge, UK; ab 2302, rabbit polyclonal, dilution 1:200). Dead cells in tumor sections were determined using the ApopTag Peroxidase In situ apoptosis detection Kit (Millipore, Billerica, MA, USA). Detection utilized the Vectastain Elite ABC kit (Vector Laboratories, Inc., Burlingame, CA, USA), and staining was visualized with DAB (Sigma-Aldrich, Steinheim, Germany). The slides were counterstained with hematoxylin, dehydrated, cleared and cover-slipped. The stained sections were imaged using an IX81 bright-field microscope (Olympus, Tokyo, Japan) and Imaging Software for Life Science Microscopy Cell. Tissue sections of each tumor were histologically examined using proliferation factor Ki67, cell death and tumor vascularization through CD31 as described [47]. For Ki67 data assessment, 20 fields per tumor were selected at random using the x40 objective and quantified using ImageJ software. For vascularization, samples were co-stained and mounted in SlowFade Gold antifade reagent with DAPI (Invitrogen, Carlsbad, CA, USA) and examined with a fluorescence microscope (Olympus IX 81, Olympus, Tokyo, Japan) with Imaging Software for Life Science Microscopy Cell.

For the volumetric measurement of lung metastases the paraffin-embedded lungs were cut into 5 µm thick sections from three different planes. The sections were stained with hematoxylin and eosin. The average sizes of metastasis were measured by computer-assisted measurement using Cellquest software (Olympus, Tokyo, Japan).

### Isolation of primary tumor cells from PyMT-induced mammary carcinomas

Primary PyMT tumor cells were isolated and cultured as described [2]. Only primary tumor cells with a low passage number (< 4 passages total) were used for experiments. All primary tumor cells were maintained in DMEM (Gibco, (Life Technologies), Paisley, UK), supplemented with 10 % fetal bovine serum (Sigma-Aldrich, St. Louise, MO, USA), 2 mM L-glutamine (Sigma-Aldrich, St. Louise, MO, USA), 20 U of penicillin and 20 µg/ml of streptomycin (Sigma-Aldrich, St. Louise, MO, USA). Cultured cells were maintained at 37°C in a humidified 5 % CO_2_ atmosphere.

### Analysis of DNA synthesis using the BrdU incorporation method

The BrdU cell proliferation kit was purchased from EMD Millipore Corporation (Billerica, MA, USA). Primary tumor cells were cultured in 96-well plates at the density of 10^5^ cells/ml. Cells were labeled with BrdU for 4 h, and levels of BrdU incorporation were determined according to the manufacturer’s instructions using a Tecan Safire (Tecan, Gröding, Austria) microplate reader at a wavelength of 450 nm and subtracting absorbance measured at 540 nm.

### Orthotopic transplantation

Primary tumor cells from three different PyMT;CstC+/+ and PyMT;CstC-/- female donors were isolated. The cells were counted, and equal amounts of cells from each animal per genotype were mixed in serum-free medium to a concentration of 7.5 million cells/ml, and 200 µl of the cell suspension was injected into the mammary fat pad of left abdominal mammary gland of 8-week-old FVB;CstC+/+ and FVB;CstC-/- recipient female mice. A total of 4 groups were established: 8 FVB;CstC+/+ mice received PyMT;CstC+/+ cells, 7 FVB;CstC-/- mice received PyMT;CstC+/+ cells, 9 FVB;CstC+/+ mice received PyMT;CstC-/- cells, and 8 FVB;CstC-/- mice received PyMT;CstC-/- cells. Palpation and a tumor volume measurement were conducted at 14 days after the tumor cell injection and continuously repeated three times a week. At 35 days after tumor cell injection, the animals were euthanized, diameters of tumors were measured, and tumors were excised and weighed.

### Cathepsin activity assay

The activity of cathepsins was detected by the hydrolysis of the fluorogenic substrate Z-Phe-Arg-4-methyl-coumarin-7-amide (Z-Phe-Arg-AMC, Bachem, Bubendorf, Switzerland). Mammary glands or primary tumors were homogenized in lysis buffer (250 mM TRIS, 10 mM EDTA 0.1 % Triton, pH 6.8), using an Ultrathurrax (IKA, Staufen, Germany). A total of 20 µg of each sample was mixed in a 96-well plate with 0.1 M phosphate buffer (1 mM EDTA, 0.1 % (v/v) PEG, 1 mM DTT, pH 6) in the presence or absence of cysteine cathepsin inhibitor E64 at a final concentration of 10 µM. Recombinant human cystatin C was produced as described [78] and was added to tumor lysates at a final concentration of 10 µM. After 15 minutes of incubation at 37 °C, substrate was added to a final concentration of 30 μM and its hydrolysis was continuously measured for 15 min using a Tecan Safire (Tecan, Gröding, Austria) plate reader at excitation and emission wavelengths of 370 and 460 nm, respectively.

When measuring the cathepsin activity in conditioned media of PyMT primary tumor cells, tumor cells of both genotypes were cultured in 12-well plates at a density of 7.5x10^5^ cells per well. The cells were incubated for 2 h at 37° C in PBS buffer (Lonza, Verviers, Belgium). After incubation, 50 µl of PBS was mixed in a 96-well plate with 0.1 M phosphate buffer, and the experiment was continued as described above.

### Immunoblotting

Equal amounts of proteins of tumor tissue or mammary glands were resolved on 12.5 % SDS-PAGE electrophoresis gels and electrotransferred to polyvinylidene difluoride membranes. The blots were probed with rabbit anti-CstC antibodies (Abcam, Cambridge, UK; ab 109508, dilution 1:5000). For the detection of different mitogen- activated protein kinases, antibodies against JNK (Cell Signaling Technology, Danvers, MA, USA; 9252, rabbit polyclonal, dilution 1:1000), phosphorylated JNK (Cell Signaling Technology, Danvers, MA, USA; 9251, rabbit polyclonal, dilution 1:1000), ERK1&2 (Abcam, Cambridge, UK; ab17942, rabbit polyclonal, dilution 1:1000), diphosphorylated ERK1&2 (Sigma-Aldrich, St Louis, MO, USA; M8159, mouse monoclonal dilution 1:10000), p38 MAPK (Cell Signaling Technology, Danvers, MA, USA; 9212, rabbit polyclonal, dilution 1:1000), phosphorylated p38 MAPK (Cell Signaling Technology, Danvers, MA, USA; 9211, rabbit polyclonal, dilution 1:1000), cathepsin L (prepared in-house; rabbit polyclonal, dilution 1:1000) [79]), cathepsin C (Santa Cruz, CA, USA; sc74590, mouse monoclonal, dilution 1:1000) cathepsin B (prepared in-house; mouse polyclonal, dilution 1:1000) [80] and legumain (Abcam, Cambridge, UK; ab183028) were used. For loading control anti-β-actin antibody (Sigma-Aldrich, St Louis, MO, USA; A1978, mouse monoclonal, dilution 1:5000) was used. Secondary antibodies were used at 1:5000 dilutions. Tissue lysates were prepared as previously described [81].

### Quantitative real-time PCR

Total RNA was isolated from flash-frozen PyMT mammary tumors with an PureLink RNA Mini kit (Ambion, Life Technologies, Foster City, CA, USA) and treated with TURBO DNA-free kit (Ambion, Life Technologies, Foster City, CA, USA) for genomic DNA elimination. RNA quality (A260/230 and A260/280 ratios) was assessed spectrophometrically, whereas RNA integrity was confirmed by 1.2% gel electrophoresis. RNA was further reverse transcribed into cDNA with a random nanomer primers using Precision nanoScript 2 Reverse Transcription kit (Primerdesign Ltd, Southampton, UK). mRNA expression levels of cathepsin B, cathepsin H, cathepsin L, cathepsin S and cathepsin X were detected by SYBR green detection chemistry using 2 X Precision MasterMix (Primerdesign Ltd). Eukaryotic translation initiation factor 4A2 (EIF4A2) was selected as the most stable expressed reference gene by geNorm reference gene selection kit (Primerdesign Ltd), and normalized values were used to calculate the relative mRNA expression of individual genes. Quantitative real-time PCR reaction was run on Mx3005P Real-time PCR system (Agilent, Stratagene products, Waldbronn, Germany) using the following conditions: 95 °C for 10 min (an initial denaturation step) followed by 40 cycles of 95 °C for 15 s and 60 °C for 1 min (annealing and extension). In addition, a melting curve (55–95 °C) was performed at the end of each run. The relative expression ratios of target genes were calculated considering their real-time PCR efficiencies. Intron spanning gene-specific primers (Primerdesign Ltd) were as follows: cathepsin B, 5’-TACTTGCTGTGGTATCCAGTGTG-3’ (forward) and 5’-ATGGTGTATGGTAAGCAGCCTAC-3’ (reverse); cathepsin H, 5’-TTTGATGTATAAAAGTGGCGTCTA-3’ (forward) and 5’-GAGTTTTTCACAATCCAGTAGAGT-3’ (reverse); cathepsin L, 5’-AAGCCATCCGTCTCTCCAG-3’ (forward) and 5’-CGTTTCTTGACAAGCCAATATTTATTC-3’ (reverse) cathepsin S, 5’- CTGTTCTTGACAAGCCAATATTTATTC-3’ (forward) and 5’-AGCCAGTAATCTTTGCCATCA-3’ (reverse); and cathepsin X, 5’-CTATGCCAATGGTCCCATCAG-3’ (forward) and 5’-CCTGGTCCTGGTGCTCAG-3’ (reverse).

### Proteomic analysis of PyMT conditioned medium

Conditioned medium of primary PyMT tumor cells was generated by 24-hour incubation of cells in serum-free medium (Gibco, (Life Technologies), Paisley, UK). The obtained conditioned medium was subsequently concentrated 100-fold using Centricon filter units with 3000 Da cut-off (Millipore, Billerica, MA, USA). The samples were separated on a 12.5 % SDS-PAGE gel (Lonza, Verviers, Belgium). The gel was stained with Coomassie Brilliant Blue, and each protein lane was cut into nine samples and destained using 25 mM NH_4_HCO_3_ (Fluka Biochemica, Buchs, Switzerland) in 50 % acetonitrile (JT Baker). Gel separated proteins were reduced with 10 mM DTT in 25 mM NH4HCO3 (Fluka Biochemica, Buchs, Switzerland), followed by alkylation with 55 mM iodoacetamide in the same buffer. Gel pieces were washed in 25 mM NH_4_HCO_3_ (Fluka Biochemica, Buchs, Switzerland) and dehydrated with acetonitrile. Trypsinization was performed overnight at 37 °C by the addition of 1 µg of trypsin (Promega, WI, Madison, USA) diluted in 80 µl of 25 mM NH_4_HCO_3_. Digested peptides were extracted from the gel with 50 % acetonitrile/ 5 % formic acid. The samples were analyzed with LTQ Orbitrap Velos (Thermo Fisher Scientific, Waltham, MA, USA) mass spectrometer coupled to a Proxeon-nanoLC. Peptides were loaded on a C18 EASY trapping column (Proxeon EASY-Column™; Thermo Fisher Scientific, Waltham, MA, USA) and separated on a C18 PicoFrit AQUASIL analytical column (New Objective, Woburn, MA, USA) at a flow rate of 300 nl/min. Elution was performed with a 90 min acetonitrile gradient from 5 % to 40 %. Nine most intense precursor ions in each full scan were selected for CID fragmentation. Dynamic exclusion was set at a repeat count of 1 with exclusion duration of 60 s. Database searches were performed with MaxQuant software, version 1.5.3.8 [82], using UniProtKB derived mouse reference proteome. The carbamidomethylation of cysteine was fixed, and the oxidation of methionine was set as a dynamic modification. Relative quantification of identified proteins was performed using spectral counting where proteins with at least 1.5-fold difference in spectral count and at least 10 detected spectra were considered as significantly different [83].

### *In vitro* cleavage of recombinant human 14-3-3β protein with recombinant human cathepsins L, S and B

Recombinant human cathepsins L, S and B were expressed in the methylotrophic yeast expression system *P. pastoris* (Invitrogen, Carlsbad, CA, USA) or in the *E. coli* expression system according to standard protocols [84, 85]. Pure mature proteins were titrated with the broad spectrum cysteine cathepsin inhibitor E-64 yielding active concentrations of 120 µM, 48 µM and 96 µM for cathepsin B, L and S, respectively. The experiment was performed in phosphate buffer, containing 100 mM Na_2_HPO_4_, 1 mM EDTA and 1 mM DTT, pH 6.0. Recombinant cathepsins were added to the 14**-**3**-**3β protein (AbFrontier, Seul, Korea) at 1:100 enzyme/substrate molar ratio. Samples were then incubated at 37°C up to 120 min. As a negative control, E64 at a final concentration of 20 µM was added to block cathepsin activity. Samples were ran on 12.5 % SDS-PAGE electrophoresis gels and silver stained as described [86].

### Statistical analysis

Quantitative data are presented as the means ± S.E. The differences were compared using Student’s t-test. Proportions were compared using the Chi-square test. When P-values were p<0.05, differences were considered statistically significant.

## SUPPLEMENTARY MATERIALS FIGURES


