# Supplementary material for: Cystatin C deficiency suppresses tumor growth in a breast cancer model through decreased proliferation of tumor cells
Source: Oncotarget. 2017 Apr 24;8(43):73793–809. doi: 10.18632/oncotarget.17379 (PMC5650301; doi:10.18632/oncotarget.17379)
Supplement: Supplementary file 1 [file oncotarget-08-73793-s001.pdf]

Cystatin C deficiency suppresses tumor growth in a breast cancer model through decreased proliferation of tumor cells

SUPPLEMENTARY MATERIALS

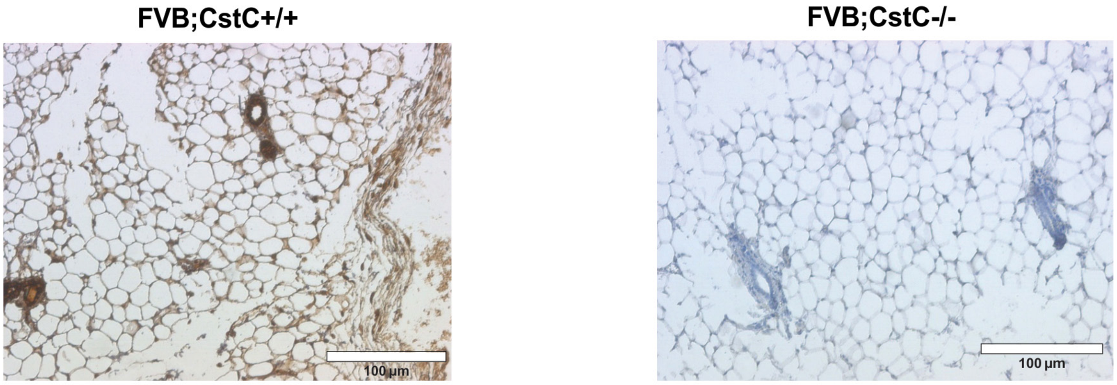

Supplementary Figure 1: Detection of cystatin C by immunohistochemistry staining of FVB;CstC+/+ and FVB;CstC-/- mammary gland sections. Scale bar, 100 µm.

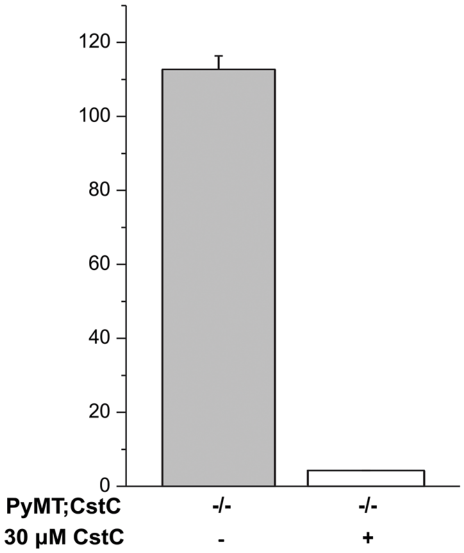

Supplementary Figure 2: Relative cathepsin activity determined in homogenates of PyMT tumors (PyMT;CstC-/- n=4). Activity was measured as Z-Phe-Arg-AMC hydrolysis in the presence or absence of human recombinant cystatin C.

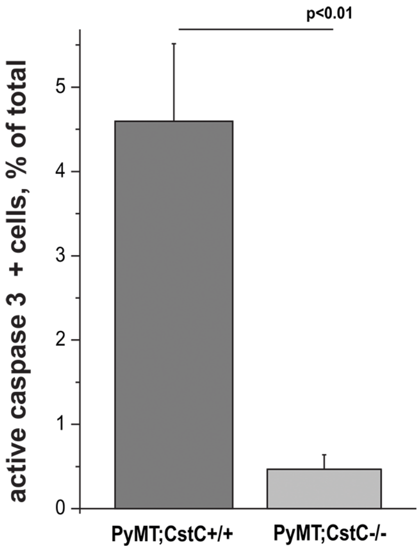

Supplementary Figure 3: Quantification of active caspase-3-positive cells as a percentage of total cells from each of PyMT;CstC+/+ (n=5) and PyMT;CstC-/- (n=5) tumors of 14 weeks old mice. Active caspase 3 + index was calculated from 5 high-power fields per tumor by computer-assisted data analysis using ImageJ software. Statistics were analyzed using Student's t-test. P<0.01.

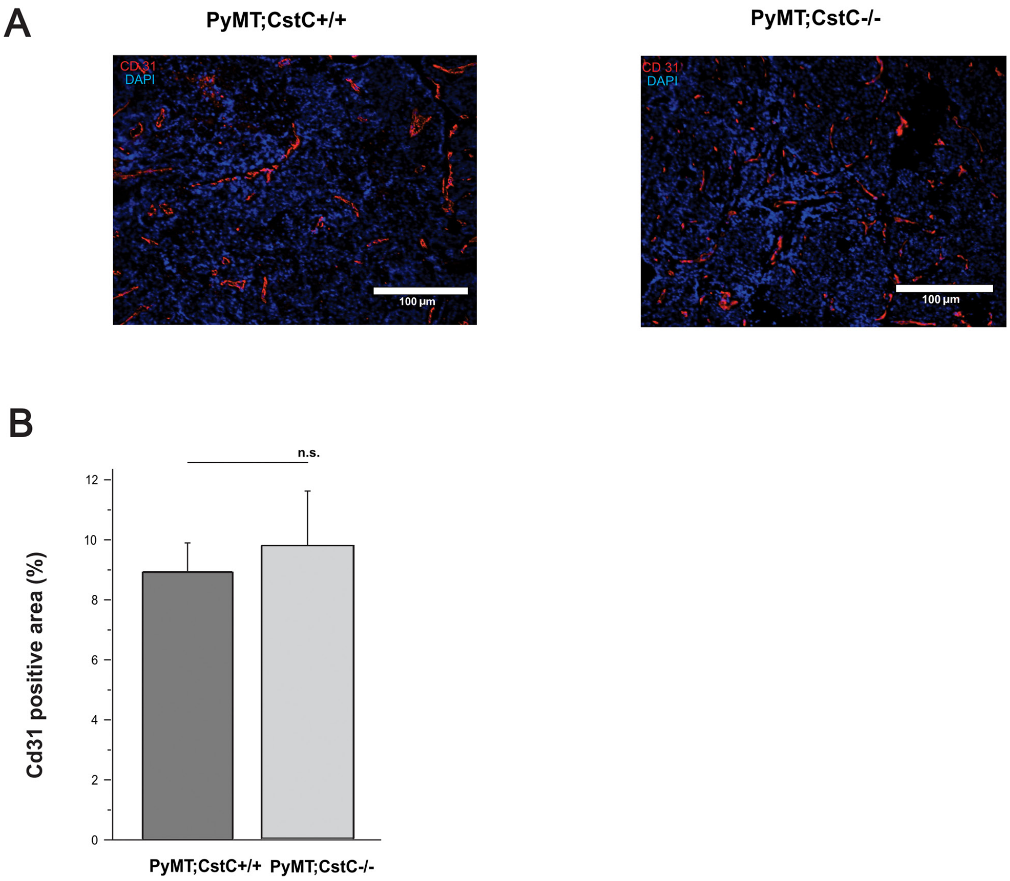

Supplementary Figure 4: (A) Representative images of immunofluorescence staining of the endothelial cell-specific marker CD31 (red staining) on PyMT;CstC+/+ and PyMT;CstC-/- tumors. (B) Quantification of CD31 positive area as percentage of all tumor area in PyMT;CstC+/+ (n=6) and PyMT;CstC-/- (n=6) tumors. Statistics were analyzed using Student`s t-test.

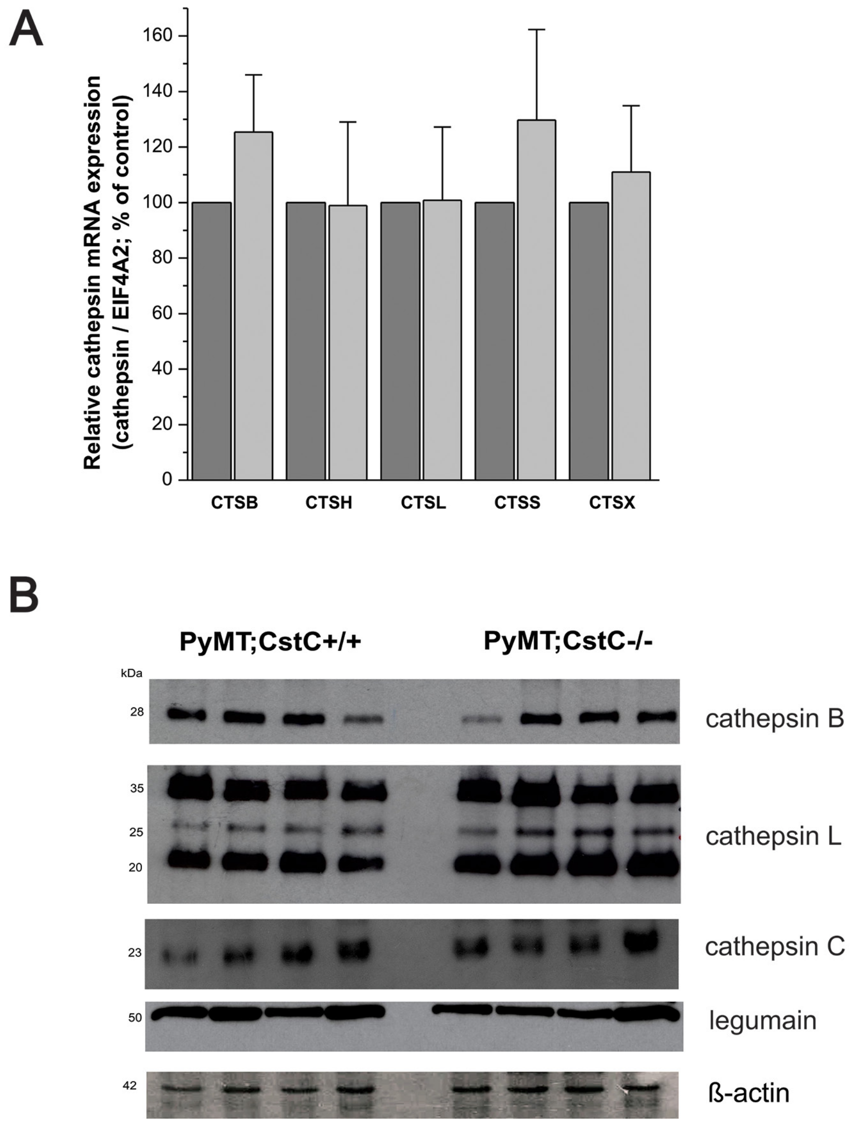

Supplementary Figure 5: (A) Expression of cysteine cathepsins in PyMT;CstC+/+ (dark gray, n=4) and PyMT;CstC-/- (light gray, n=4) in primary mammary tumors. Comparison of mRNA expression of cathepsin B (CTSB), cathepsin H (CTSH), cathepsin L (CTSL), cathepsin S (CTSS) and cathepsin X (CTSZ) in tumor samples was measured by quantitative real-time PCR. Data are presented as means and s.e., expressed as a percentage of the control (100%). **(B)** Western blot analysis of cathepsin B, cathepsin L, cathepsin C and legumain in lysates of representative tumors from 4 PyMT;CytC+/+ and 4 PyMT;CstC-/- mice. β-actin served as a loading control.

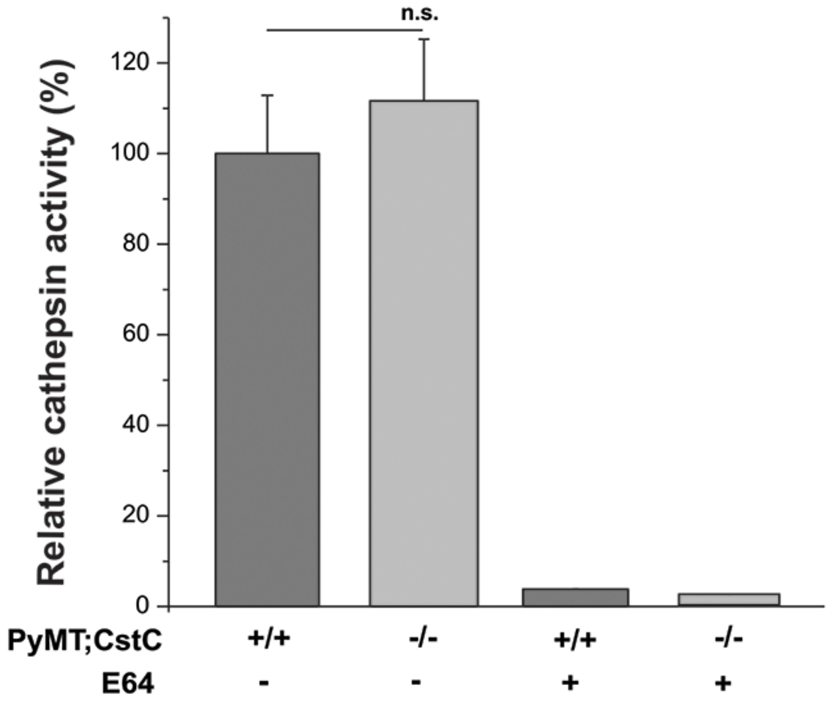

Supplementary Figure 6: (A) Relative cathepsin activity determined in homogenates of primary tumor cells (PyMT;CstC+/+ n=3 and PyMT;CstC-/- n=3). Activity was measured as Z-Phe-Arg-AMC hydrolysis in the presence or absence of E64. Statistics were analyzed using Student`s t-test.
